# Muscle Invasive Bladder Cancer: From Diagnosis to Survivorship

**DOI:** 10.1155/2012/142135

**Published:** 2012-08-10

**Authors:** N. E. Mohamed, M. A. Diefenbach, H. H. Goltz, C. T. Lee, D. Latini, M. Kowalkowski, C. Philips, W. Hassan, S. J. Hall

**Affiliations:** ^1^Department of Urology and Oncological Science, Mount Sinai School of Medicine, New York, NY 10029-6574, USA; ^2^Michael E. DeBakey VA Medical Center, University of Houston-Downtown and Baylor College of Medicine, Houston, TX 77030-4298, USA; ^3^Department of Urology, University of Michigan Comprehensive Cancer Center, Ann Arbor, MI 48109-0944, USA; ^4^Department of Urology, Tawam Hospital, 15258 Al Ain, UAE

## Abstract

Bladder cancer is the fifth most commonly diagnosed cancer and the most expensive adult cancer in average healthcare costs incurred per patient in the USA. However, little is known about factors influencing patients' treatment decisions, quality of life, and responses to treatment impairments. The main focus of this paper is to better understand the impact of muscle invasive bladder cancer on patient quality of life and its added implications for primary caregivers and healthcare providers. In this paper, we discuss treatment options, side effects, and challenges that patients and family caregivers face in different phases along the disease trajectory and further identify crucial areas of needed research.

## 1. Introduction

Cancer diagnosis is often perceived as a traumatic event that changes an individual's basic assumptions about the self as effective and powerful, and the world as benevolent, controllable, and predictable [1, 2]. This event is even more devastating when cancer patients undergo extensive surgeries that severely debilitate their body image and their psychological and social well-being. Muscle-invasive bladder cancer (MIBC) provides a powerful, yet understudied example of the impact that cancer diagnosis and treatments may have on patients' emotional, physical, functional, and social adjustment [3].

Previous research in this patient population has primarily been focused on health-related quality of life (HRQOL) as an indicator of posttreatment adjustment [4]. Yet, in addition to decrements in HRQOL and body image, patients with MIBC must often resolve treatment decision-related conflicts and overcome financial, communication, literacy, and healthcare service barriers as they move through different phases of the cancer trajectory (i.e., cancer diagnosis, treatment, and survivorship). Despite treatment-related physical and social limitations, patients need to: (1) balance the uncertainty associated with diagnosis and the risk of recurrence; (2) maintain previous social roles and functions; (3) solve financial issues arising from insurance issues and uncertain employment situations.

In this paper, we will provide a comprehensive overview of: (1) MIBC incidence, mortality rates, and risk factors; (2) MIBC-treatment-related side effects; (3) challenges that newly diagnosed MIBC patients face during treatment decision making, treatment-related side effects, and posttreatment healthcare; (4) indicators of posttreatment adjustment survivorship issues. We will address limitations in the existing literature and discuss future directions.

## 2. Radical Cystectomy

MIBC may be treated with curative intent by either external beam radiation therapy (with or without chemotherapy) or radical cystectomy, with or without perioperative chemotherapy [5]. In the United States, radical surgery is considered standard of care. Radical cystectomy also remains the treatment of choice for NMIBC refractory to transurethral resection and intravesical therapy, as well as invasive carcinoma of the prostatic urethra. In men, radical surgery traditionally includes the removal of the bladder, prostate, and seminal vesicles; in women the urethra, uterus, cervix, ovaries, fallopian tubes, and anterior vagina have historically been removed along with the bladder [6–10]. In the recent past, however, strategies to preserve sexual and reproductive function in selected patients have led to prostate capsule and seminal vesicle sparing surgery in men and to vaginal sparing surgeries in women with preservation of the reproductive organs based on the patient's fertility status. Radical cystectomy necessitates urinary diversion using one of three major methods: incontinent cutaneous (e.g., the ileal conduit), continent cutaneous, or orthotopic (e.g., the neobladder) diversion.

### 2.1. Incontinent Cutaneous Diversion

Incontinent cutaneous diversion optimal in a wide spectrum of patients. Conduits can be created using large or small intestine, but due to the manageable metabolic profile and the ease of the bowel segment, the ileal conduit is performed most commonly. An ileal conduit is an incontinent urinary diversion to the skin, requiring the creation of a cutaneous stoma and use of external stoma appliances to collect urine. An ileal conduit is considered the easiest urinary diversion to be performed surgically. Despite the fact that this is the diversion of choice for patients with hepatic or renal insufficiency or those unable to catheterize or timed void, this method has its drawbacks. Patients reporting early and late complications for ileal conduit range from 18% to 30% [11]. Stoma-related complications are common and have been reported in up to 31% of patients; these include parastomal hernia, urinary leakage, and stomal stenosis [11]. Urinary tract deterioration was found in 35% after 5-year followup and in 50% after 15-year followup. Skin irritations due to direct and continuous contact with urine, as well as constipation were also reported [7, 9].

### 2.2. Continent Cutaneous Diversion

This method involves creating an intra-abdominal, catheterizable urinary reservoir commonly using up to 60–70 cm of ileum, the entire right colon or a combination of small and large bowel. The continent mechanism varies based on the surgical approach [12]. The continent reservoir requires the use of a catheter to drain urine from the reservoir four to six times per day. Failure to catheterize as frequently as recommended can cause serious medical conditions including acute renal failure, perforation, and infection [13]. Early and late complication rates for continent reservoir range from 3%–7% and from 13%–30%, respectively [14]. Physical morbidity reported after continent urinary diversion includes stenosis, pouch leakage, pouch rupture (1.5% to 4.3%) [13], and incontinence. Overall, 3.2%–7.4% of patients report these complications [15]. Additionally, night-time catheterization may be more bothersome than night-time stoma care associated with ileal conduits [16].

### 2.3. Orthotopic Diversion

This procedure involves the creation of a large-capacity, low-pressure reservoir from colon or ileum that is connected to the native urethra [11]. Patients learn to void through simultaneous relaxation of the pelvic muscles with coordinated increase in their intra-abdominal pressure (Valsalva maneuver) [12, 16, 17]. Clean intermittent catheterization (CIC) is required to manage urinary retention or periodically for irrigation of excessive mucous [12, 16, 17]. Patients who are not able to catheterize themselves practice timed voiding (since normal bladder sensation and urge are no longer intact). Patients with hepatic or renal insufficiency are generally not eligible for this procedure. The time needed for postoperative recovery and for achieving urinary continence is longer in older than in younger patients [16, 17]. Additionally, patients with prostatic stromal or urethral involvement and many women with bladder neck disease are not eligible for this procedure since the urethra must be removed for adequate cancer control. Daytime continence ranges from 67% to 100% and night-time continence ranges from 45% to 65% [12, 18]. The high-storage capacity and interaction of urine with the absorptive surface of the bowel continent reservoir and neobladder can ultimately cause metabolic disturbances and renal failure [11].

## 3. Psychosocial Effects of Urinary Diversion

### 3.1. Ileal Conduit

The emotional, functional, physical, and social impact of radical surgery on HRQOL significantly varies by the urinary diversion form [4, 7, 9, 12]. Studies have shown that ileal conduits are considered the easiest urinary diversion to perform surgically; however, [15] it is associated with significant psychological and practical side effects that can drastically reduce the emotional well-being of the patient [7, 9]. These effects include: (1) changed social roles decreased body image, sexual dysfunction, and barriers due to body image, bother caused by urinary leakage, odor, and frequent stoma care; (2) difficulties in activities of daily living and social activities, such as, bathing and sleeping; (3) barriers to participation in previously pleasurable hobbies and travelling [7, 9, 15]. Studies that examined bladder and bowel cancer patients' coping with ostomy surgeries showed that feelings of shock, hate, disgust, repulsion, embarrassment, devastation, and unacceptance were common [19–21]. Patients' coping with ileal conduit and its consequences may depend largely on the patient's personal and social resources and the meaning and importance placed on body image, sexuality, and urinary control [19–21]. Adequate explanation of the treatment's potential effects on sexual function, urinary control, and body image should be provided by the physician during counseling.

### 3.2. Continent Reservoir

In patients treated with continent reservoir, the body image, theoretically, is less disturbed and stoma care is generally not required as compared to incontinent diversion, but failure to catheterize as frequently as needed can cause urinary leakage and serious medical conditions [22]. In addition, night-time catheterization may result in reduced amount and quality of sleep [21]. In spite of the reduced impact of a continent reservoir on the patient's body image (compared to ileal conduit), the need for regular catheterization may limit the patient's activities of daily living and his/her participation in work and social activities. Moreover, dietary modifications to avoid frequent urostomy appliance management, voiding, or catheterization required with incontinent and continent reservoirs may result in increased risk for serious medical conditions including acute renal failure [22]. These complications could further debilitate the patient's QOL and emotional adjustment.

## 4. Neobladder Diversion

A major advantage of the neobladder is that it preserves body image and may be socially desirable more than the ileal conduit and continent reservoir, as it maintains urethral voiding [3]. Patients with neobladder diversion scored better in a scale assessing posttreatment social and physical activities including bathing, sleeping, and travelling and reported fewer sexual problems and barriers than patients with ileal conduit [4]. Although daytime continence is achieved in almost all patients with neobladder diversion, night-time continence is less likely to be achieved. Results from recent research in other patient populations demonstrated the negative impact of night-time incontinence on increased depression and poor quality of life [23, 24]. Additionally, there is evidence that physical impacts of neobladder diversion on QOL vary by gender [18]. Urinary incontinence and deterioration of sexual function are more common among women compared to men following surgery and orthotopic neobladder [18]. Follow-up clinic assessments should examine emotional wellbeing and sexual function among patients regardless of the type of urinary diversion received.

### 4.1. Cancer Recurrence

The risk of local recurrence following cystectomy has decreased markedly from about 40% reported in initial reports to 6% to 13% observed in recent findings [23, 24]. Approximately 50% of MIBC patients relapse with distant recurrence [9, 25]. The probability of cancer recurrence is associated with tumor stage, grade, and node status at the time of cystectomy. The high rate of recurrence and the concern for disease progression in patients with MIBC necessitate careful surveillance following cystectomy (i.e., every 3–6 months in the first year, every 6 months in the second year and annually thereafter).

### 4.2. Posttreatment Self-Care

Patients and primary caregivers (referred to as family caregivers throughout this paper) are required to manage the urinary diversion, which involves the acquisition of new skill sets, based upon the diversion choice. Patients with ileal conduit diversion are instructed in the use of stomal appliances and skin care beginning on the second or third day following surgery [26]. The education continues, however, throughout the first several months after diversion as a variety of stomal supplies are explored to provide the best fit for the individual's changing habitus and activity level. For patients with continent diversions and neobladders, training occurs in the first postoperative visits (i.e., 2-3 weeks postsurgery). At this time, catheters and/or drains are removed as patients become more able to manage their pouch. Patients are then instructed to empty the neobladder (through Valsalva voiding or intermittent catheterization) or continent reservoir (via catheterization) on a timed schedule that will permit an increasing pouch capacity [15, 17].

The American Urological Association (AUA) and the Wound Ostomy Continence Nurse Society (WOCN) Joint Position Statement on the Value of Pre-operative Stoma Marking for Patients Undergoing Urinary Ostomy Surgery recommends that patients scheduled to have cystectomy learn about stoma care and use of stoma appliances prior to surgery when they are less distracted than in the immediate postoperative period as typically implemented in BC health care settings [24]. The Joint Position Statement, however, lacks recommendations for patients undergoing continent urinary diversion [24]. It is certainly advantageous for patients with planned continent diversions to gain greater insight into the postoperative management of their diversion prior to treatment. Informing the patient and the family caregiver about treatment effects, emphasizing the importance of timed voiding and self-catheterizing, and providing the skills needed for postsurgical health care before treatment may reduce the risk of posttreatment complications related to voiding habits and enhance treatment decision making and posttreatment QOL.

### 4.3. Treatment Decision and Decision-Related Outcomes

There is no consensus on the best diversion choice for patients presenting with MIBC as each of the three previously mentioned diversion methods has clear advantages and disadvantages as discussed in the previous sections [4, 7, 9, 11, 13]. Current data on the long-term efficacy of various treatment options are sparse, and support for these techniques is derived primarily from their short- to intermediate-term functional results [11]. This situation can potentially increase the difficulty in making treatment decisions as newly diagnosed patients must consider incomplete data about treatment options and side effects, as well as contradictory treatment recommendations from their physicians. Results of semistructured patient interviews revealed that patients often lack full information about treatment options and related side effects, depending on their physician for this information, resulting in decreased participation in decision making [26]. Patients need to understand how differing treatment options might influence their QOL in the short and long term when making treatment decisions. In addition, physicians need to be able to objectively communicate treatment and side-effect information to patients and facilitate integration of this information into their patients' decision-making process [27, 28]. Incomplete information about treatment, its risks and benefits, and its impact on lifestyle and relationships may cause patients to make treatment decisions incompatible with their values and preferences and lead to dissatisfaction with treatment and a decline in patient care [27, 28]. When compared to other cancers, literature on BC treatment decision making is scarce.

## 5. Indicators of Posttreatment Adjustment: ****Health-Related Quality of Life

Most of the research on the adjustment to a diagnosis of BC and its treatment has focused on examining perceived HRQOL following treatment. Perceived HRQOL refers to patients' appraisal of and satisfaction with their current level of physical, emotional, and social functioning as compared to what they perceive to be possible or ideal [28]. In the case of patients subjected to radical cystectomy and urinary diversion, posttreatment QOL and skills needed for posttreatment health care could significantly affect the patient's choice of the type of urinary diversion form as no significant differences have been determined between the three urinary diversion forms in terms of cancer control and survival rate [11, 29, 30]. In spite of the increased research focus on QOL following cystectomy and urinary diversion, little is known about the psychosocial impact of treatment for MIBC.

### 5.1. Measures of Quality of Life Following Invasive Bladder Cancer Treatment

HRQOL among MIBC patients is commonly assessed using generic measures, disease-specific measures, or both. Generic HRQOL measures typically assess emotional and functional well-being; examples include *the profile of mood state*, the medical outcome study's 36-item *short form health survey* (sf-36), and *the quality of wellbeing scale* [13, 31]. Although generic measures allow for easy comparisons of HRQOL between different diseases and treatment types and are commonly used among cancer patients, they are not sensitive to bladder cancer treatment-specific side effects [9]. This lack of sensitivity to treatment-specific side effects may limit their ability to detect important aspects of BC-related HRQOL and potential changes in posttreatment HRQOL [9].

Disease-specific HRQOL measures are used to assess broad cancer treatment side effects or BC-specific effects (see Table 1 for more examples). Measures assessing general cancer effects include The European Organization for Research and Treatment of Cancer (EORTC),* Quality of Life Questionnaire version 3.0 QLQ-C30* (EORTC QLQ-C30), and *The Functional Assessment of Cancer Therapy-General* (FACT-G) [13, 31]. Those assessing MIBC effects include the EORTC's Quality of Life Questionnaire QLQ-BLM30 (EORTC-QLQ-BLM30) and *The Functional Assessment of Cancer Therapy-Bladder *(FACT-BL) [13, 31]. Although, these scales are more sensitive to changes in HRQOL following BC treatment, they lack sensitivity to specific psychological impact of treatment and gender differences in treatment side effects and differences in side effects between urinary diversion options [9]. Additionally it is important to address patients' beliefs (i.e., identity, timeline, cure beliefs, consequences) and expectations regarding their treatment outcomes [32] Applications of the self-regulation theory (SRT) in cancer research showed patient illness perceptions and expectations that can influence posttreatment emotional adjustment directly and indirectly by influencing patients' control beliefs and coping strategies [1, 2]. There is a need for research and outcomes measures that address these issues among patients with MIBC.

### 5.2. Quality of Life Research Outcomes in Invasive Bladder Cancer Patients: Methodological Issues

 The international medical literature on QOL following MIBC treatment focused on examining global QOL as well as disease-specific HRQOL (see Table 2). Although no superiority of any of the three urinary diversion forms in terms of overall QOL was found, patients with ileal conduits reported more problems with stomal issues, such as, leakage and bother with odor, less sexual activities, less satisfaction with treatment outcomes, greater distress with urine leakage, and lower body image compared to patients treated with cystectomy and continent reservoir [6, 33–38]. Although, in some studies, patients with neobladders scored better in physical function and body image than those with ileal conduit and continent reservoirs, they were also more likely to report a general decrease in urinary function and higher incontinence rates than those with ileal conduit and continent reservoirs [37, 39–42]. In spite of the preponderance of studies that examined QOL in MIBC patients, the methodological limitations of these studies may limit the generalizability of their outcomes [4, 29, 43]. These methodological limitations include lack of longitudinal and prospective research that examined changes in posttreatment HRQOL and the paucity of randomized controlled studies testing the validity and reliability of tools used in the assessment of HRQOL [4, 29, 43]. Given these methodological limitations, questions remain as to the nature of the information HRQOL research provides and whether this information is of clinical use during preoperative consultations with patients. Table 2 depicts examples of studies and variations in HRQOL measures in patients undergoing radical surgery.

## 6. Indicators of Posttreatment Adjustment: ****Impact of Age and Overall Health

After age 30, most organ systems begin exhibiting progressive changes in physiological functioning, even in the absence of disease [39]. These changes result in declines in cognitive, emotional, physical, and social functioning status and affect roughly 60% and 76% of adults ages 65–79 and 80 years and older, respectively. A recent cohort study of older BC patients found that comorbidities, such as, heart disease, stroke, arthritis, and urinary, hearing, and vision problems increased in prevalence with age in this population [40].

Current health status and functioning are often key factors in determining eligibility for cystectomy and other MIBC treatments. For example, much older adults are less likely to undergo radical cystectomy. Rates of 55% have been reported for adults ages 55–59 years compared to 24% and 16% for those ages 75–79 years and 80–84 years [40]. Additionally, when older adults are offered cystectomy, they are typically offered ileal conduits and not neobladders [39]. They are also less likely to receive optimal doses of chemotherapeutic agents [39].

Thus, older adults diagnosed with MIBC face twin challenges to maintaining good quality of life. These include (1) addressing the effects of physiological aging and comorbidities, which may reduce their eligibility for and tolerance of invasive BC treatments, such as, cystectomy or chemotherapy; (2) addressing the impact of treatment-related comorbidities and postsurgical complications. Various studies report perioperative morbidity rates of 30–60% among older adults [39]. However, chronological age may not be predictive of the severity of such complications. One study found differences in overall complication rates for older adults receiving neobladders (versus ileal conduits) while another found no differences in daytime urinary continence [39]. Nighttime continence was almost 100% at 5-year followup for adults 50 years or younger and 90% for those over age 60. Beyond urinary continence, few studies have focused on other quality of life issues among postcystectomy older adults, such as, sexual functioning, psychological functioning, social relationships, body image, and gender roles.

## 7. Indicators of Posttreatment Adjustment: ****Psychological Distress

Cancer diagnosis may be considered the archetypal experience of loss and is a continuous threat to the patient's life. Cancer does not only signify an existential plight; it may arouse extreme negative emotions among those who are affected [2]. Accordingly, patients suffering from cancer or other life-threatening diseases usually need to change goals and disengage from many commitments in order to cope with the multiple medical, social, psychological and financial implications of their conditions. Although previous research among certain cancer populations has examined psychological distress, depression, coping, and emotional adjustment in both newly diagnosed patients and survivors, these issues are essentially unexplored in patients with MIBC [2, 41].

## 8. Survivorship Issues of MIBC Patients ****and Their Family Caregivers

Examining needs among cancer survivors and their family caregivers revealed different areas of unmet needs [2, 42]. These include: psychological needs (i.e., needs for help with emotional issues), health system and information needs, physical and social daily living needs, emotional support, and interpersonal communication needs [66]. Family caregivers of cancer survivors often feel unprepared for the cancer experience, have limited knowledge about what to expect regarding cancer and treatment, and receive little guidance and support from the oncology team about how to provide care and support to the patient during and following treatment [67, 68]. Moreover, among younger and middle-aged family caregivers, worries about job loss, other family responsibilities, limited social activities of daily living, and reduced productivity add to the burden of care giving [66, 68, 69]. Most family caregivers of BC patients, however, are older. This population is especially vulnerable to the emotional and physical impact of caregiving, particularly in the face of their own health problems and limited economical and social resources when compared to younger family caregivers. Older family caregivers are more likely to have comorbid diseases, live on a fixed income, and have a limited social network while they provide care and support for the patient. As a result, older family caregivers are more likely to become fatigued from interrupted night sleep and the emotional burden of caregiving. Poor physical condition, increased depressive symptoms, and greater mortality are risks encountered among older family caregivers [69, 70]. To date, no study has examined determinants of psychological adjustment and needs of BC patient and their family caregiver across various types of urinary diversion.

## 9. Implications for Practitioners ****and Researchers

Given the side effects of radical surgical treatment for MIBC, there is a strong need for consistent and clear communication between care providers and patients/family caregivers. Increasing patient awareness about treatment options, associated risks and benefits, and short-and long-term treatment effects may aid the patient's decision making and postoperative preparedness. More consistent use of HRQOL instruments in urologic practice, assessment of family caregivers support, and pre- and postsurgical counseling can facilitate patients' coping and satisfaction. More research is needed to examine both patient and family caregiver needs in the context of age, gender, and diversion type. Identifying these needs preoperatively is vital in providing necessary support to reduce care burden and to ultimately guide the design and evaluation of future psychosocial interventions.

## 10. Conclusions

MIBC presents challenges to both the patient and the family caregiver at each step along the disease trajectory; from diagnosis to treatment to survivorship. Patients diagnosed with MIBC are expected to live for the rest of their lives with the physical emotional and social consequences and practical implications of their treatment choices. Consequently, it is important for patients and the family caregivers to understand how different treatment options might influence their quality of life in the short- and the long-term when making these decisions, particularly concerning the urinary diversion. However, the optimal form of urinary diversion is uncertain. Patients and family caregivers need to evaluate imperfect data from various studies and balance perceived benefits of a diversion with the reality of reduced quality of life and added burden of health. The difficulty of making a treatment decision is further compounded by the small window of time between diagnosis and treatment which make seeking 2nd and 3rd experts' opinions a challenge. Despite the gravity of treatment and the associated side effects and posttreatment care, no study has examined the needs of MIBC survivors and their family caregivers. A proactive approach should be undertaken to prepare the patient and the family caregiver for posttreatment health management, prior to therapy. Such action may facilitate patient decision making and also assist both the patient and family caregiver in coping with difficulties and challenges that arise after treatment.

## Figures and Tables

**Table 1:** Quality-of-life instruments. (With permission from Lee et al. [44]).

Instrument	Institution	Domains	Validated	Reliable	Cancer specific	Bladder cancer specific	No. of items
BCI [33, 45]	University of Michigan	Urinary Bowel Sexual function	Yes	Yes	Yes	Yes	34
FACT-VCI [46]	Vanderbilt University	See FACT-G Urinary Bowel Sexual function	Yes	Yes	Yes	Yes (limited tocystectomy)	45
QLQ-BLM 30 module [47]	EORTC	Urinary Bowel Sexual function	Ongoing studies	Ongoing studies	Yes	Yes (muscle-invasive disease)	30
QLQ-BLS 24 Module [45]	EORTC	Urinary Bowel Sexual function	Ongoing studies	Ongoing studies	Yes	Yes (nonmuscle-invasive disease)	24
FACT-BL [48]	FACIT	See FACT-G Limited urinary Limited bowel Limited sexual function	Ongoing studies	Ongoing studies	Yes	Yes	39

FACT-G [49]	FACIT	Physical Social Emotion Function	Yes	Yes	Yes	No	27
QLQ-C30 [47]	EORTC	5 Functional scales 3 Symptom scales 1 Global health/QOL scale	Yes	Yes	Yes	No	30

SF-36 [34]	RAND	8 Domains including Physical Mental Social function Emotional	Yes	Yes	No	No	36

BCI: Bladder Cancer Index; EORTC: European Organization for the Research and Treatment of Cancer.

FACIT: The Functional Assessment of Chronic Illness Therapy.

FACT-BL: Functional Assessment of Cancer Therapy—Bladder Cancer (Extension of the FACT-G + 12 additional bladder cancer-specific items including incontinence, diarrhea, body image, sexual function, and stoma care).

FACT-G: Functional Assessment of Cancer Therapy General.

FACT-VCI: Functional Assessment of Cancer Therapy—Vanderbilt Cystectomy Index (Extension of the FACT-G + 17 additional bladder cancer and treatment specific items).

QLQ-C30: EORTC Quality-of-Life Core Questionnaire.

QLQ-BLM30: EORTC Quality-of-Life Core Questionnaire—Bladder Cancer Muscle Invasive (Extension of the QLQ-C30 + 30 additional bladder cancer-specific items; http://www.eortc.be/home/qol/files/SCManualQLQ-C30.pdf).

QLQ-BLS24: EORTC Quality-of-Life Core Questionnaire—Bladder Cancer Superficial (Extension of the QLQ-C30 + 24 additional bladder cancer-specific items; http://www.eortc.be/home/qol/files/SCManualQLQ-C30.pdf).

RAND Corporation: **R**esearch **AN**d Development.

SF-36: Medical Outcomes Study 36-Item Short Form.

**Table 2:** Quality of life in patients treated with continent and incontinent urinary diversion.

Author (Year)	Location	Sample size			Scale(s) used	Principal findings
		IC	CCD	NB		
Prospective longitudinal studies of quality of life in patients treated with urinary diversion						

Hardt et al. [8] (2000)	Germany	24	20		SF-36, Self-design	Sexual fxn similar, all SF-36 domains returned to baseline by 1yr except physical fxn. Note: 75% would choose the same diversion
Somani et al. [50] (2009)	UK	29		3	SEIQoL-DW, SWLS, EORTC QLQ-C30	“Family”, “health”, “relationships”, and “finance” identified as biggest contributors to QoL

Cross-sectional studies of quality of life in patients treated with urinary diversion						

Boyd et al. [35] (1987)	USA	87	85		BDI, POMS, Self-design	Preoperative education important for adaptation. CCD: more likely to be sexually active
Mansson et al. [51] (1988)	Sweden	40	20		Self-design	IC: more problems with stomal issues such as leakage + odor
Bjerre et al. [52] (1995)	Denmark	29		38	Self-design	NB with greater incontinence, but IC with greater distress from leakage and lower body image
Gerharz et al. [36] (1997)	Germany	131	61		Self-design	Similar coping strategies, social support. CCD: Better global QOL and fewer stomal issues
Okada et al. [6] (1997)	Japan	63	74		Self-design	CCD: less local stoma problems; greater satisfaction
Hart et al. [37] (1999)	USA	25	93	103	4 Self-report questionnaires	Overall, high QOL in all groups. IC: worse social function
Kitamura et al. [53] (1999)	Japan	36	22	21	EORTC QLQ-C30	Similar overall QOL between groups. IC: more trouble with public restrooms + travel
Fujisawa et al. [39] (2000)	Japan	20	36		SF-36	Similar QOL between diversion types
McGuire et al. [54] (2000)	USA	38	16	38	SF-36	IC: statistically lower mental well being than population-based norm.
Conde Redondo et al. [55] (2001)	Spain	6		27	Self-design	IC: greater distress with urine leakage + lower body image
Hobisch et al. [56] (2001)	Austria	33		69	EORTC QLQ-C30	NB better across all domains
Dutta et al. [57] (2002)	USA	23		49	SF-36, FACT-G	NB marginally better on several domains

Cross-sectional studies of quality of life in patients treated with urinary diversion						

Hara et al. [58] (2002)	Japan	37		48	SF-36, Self-design	Equivalent in all domains of SF-36
Mansson et al. [59] (2002)	Sweden		35	29	FACT-BL, HADS	NB: more problems with incontinence
Protogerou et al. [60] (2004)	Greece	58		50	EORTC QLQ-C30	Included matched 54 patient control group. IC with greater urine odor and day and nighttime leakage than NB or controls.
Allareddy et al. [61] (2006)	USA	56	26		FACT-BL	Compared to intact bladder (*n* = 177): Sexual fxn lower in individuals undergoing cystectomy. Similar QOL between diversion groups.
Kikuchi et al. [40] (2006)	Japan	20	14	15	FACT-BL	Overall QOL similar between groups. NB: worse urinary control, better body image
Gilbert et al. [33] (2007)	USA	66		122	BCI	Urinary, bowel and sexual differences exist by diversion type. NB may have worse urinary fxn
Saika et al. [62] (2007)	Japan	56	31	22	EORTC QLQ-C30, Satisfaction	Assessment of patients 75+ yrs old: Similar QOL between diversion types, including 31 with ureterocutaneostomy.
Harano et al. [63] (2007)	Japan	20		21	SF-36, Self-design	Similar QOL between diversion types
Philip et al. [64] (2009)	United Kingdom	24		28	SF-36	Overall QOL similar NB: Better physical function
Sogni et al. [65] (2008)	Italy	18		16	EORTC: QLQ-C30, QLQ-BLM30	Assessment of patients 75+ yrs old: Similar QOL between diversion types. NB: 56% and 25% daytime and nighttime complete continence rates
Hedgepeth et al. [19] (2010)	USA	89		144	BCI, EORTC BIS	Included bladder-intact comparison group (*n* = 112); NB: Lower urinary function. Body Image is similar between NB and IC.

Note. Urinary Diversion Type: IC: Ileal conduit, CD: Continent cutaneous diversion, NB: Orthotopic Neobladder.

“Self-design”: questionnaires developed specifically for the study.

SF-36: Medical Outcomes Study 36-Item Short Form.

HADS: Hospital Anxiety and Depression Scale.

EORTC BIS: European Organization for the Research and Treatment of Cancer Body Image Scale.

SEIQoL-DW: Schedule for Evaluation of Individual Quality of Life-Direct Weighting.

SWLS: Satisfaction With Life Scale.

BDI: Beck Depressive Inventory.

POMS: The Profile of Mood States.

## References

[1] Diefenbach M, Mohamed NE, Horwitz E, Pollack A (2008). Longitudinal associations among quality of life and its predictors in patients treated for prostate cancer: the moderating role of age. *Psychology, Health and Medicine*.

[2] Janoff-Bulman R (1989). Assumptive worlds and the stress of traumatic events: applications of the schema construct. *Social Cognition*.

[3] Kulaksizoglu H, Toktas G, Kulaksizoglu IB, Aglamis E, Unluer E (2002). When should quality of life be measured after radical cystectomy?. *European Urology*.

[4] Wright JL, Porter MP (2007). Quality-of-life assessment in patients with bladder cancer. *Nature Clinical Practice Urology*.

[5] Pectasides D, Pectasides M, Nikolaou M (2005). Adjuvant and neoadjuvant chemotherapy in muscle invasive bladder cancer: literature review. *European Urology*.

[6] Okada Y, Oishi K, Shichiri Y (1997). Quality of life survey of urinary diversion patients: comparison of continent urinary diversion versus ileal conduit. *International Journal of Urology*.

[7] Turner WH, Studer UE (1997). Cystectomy and urinary diversion. *Seminars in Surgical Oncology*.

[8] Hardt J, Filipas D, Hohenfellner R, Egle UT (2000). Quality of life in patients with bladder carcinoma after cystectomy: first results of a prospective study. *Quality of Life Research*.

[9] Stein JP, Lieskovsky G, Cote R (2001). Radical cystectomy in the treatment of invasive bladder cancer: long-term results in 1,054 patients. *Journal of Clinical Oncology*.

[10] Clark PE (2002). Urinary diversion after radical cystectomy. *Current Treatment Options in Oncology*.

[11] Krupski T, Theodorescu D (2001). Orthotopic neobladder following cystectomy: indications, management, and outcomes. *Journal of Wound, Ostomy and Continence Nursing*.

[12] Walsh PC, Wein AJ, Vaughan ED, Retik AB, Peters CA (2002). *Cambell Urology*.

[13] Botteman MF, Pashos CL, Hauser RS, Laskin BL, Redaelli A (2003). Quality of life aspects of bladder cancer: a review of the literature. *Quality of Life Research*.

[14] Benson MC, Olsson CA (1999). Continent urinary diversion. *Urologic Clinics of North America*.

[15] Filipas D, Egle UT, Budenbender C (1997). Quality of life and health in patients with urinary diversion: a comparison of incontinent versus continent urinary diversion. *European Urology*.

[16] Studer UE, Danuser H, Hochreiter W, Springer JP, Turner WH, Zingg EJ (1996). Summary of 10 years’ experience with an ileal low-pressure bladder substitute combined with an afferent tubular isoperistaltic segment. *World Journal of Urology*.

[17] Stein JP, Penson DF, Lee C, Cai J, Miranda G, Skinner DG (2009). Long-term oncological outcomes in women undergoing radical cystectomy and orthotopic diversion for bladder cancer. *Journal of Urology*.

[18] Lantz AG, Saltel ME, Cagiannos I (2010). Renal and functional outcomes following cystectomy and neobladder reconstruction. *Journal of the Canadian Urological Association*.

[19] Hedgepeth RC, Gilbert SM, He C, Lee CT, Wood DP (2010). Body image and bladder cancer specific quality of life in patients with ileal conduit and neobladder urinary diversions. *Urology*.

[20] Brown H, Randle J (2005). Living with a stoma: a review of the literature. *Journal of Clinical Nursing*.

[21] Hurny C, Holland J (1985). Psychosocial sequelae of ostomies in cancer patients. *Cancer Journal for Clinicians*.

[22] Colombo R, Naspro R (2010). Ileal conduit as the standard for urinary diversion after radical cystectomy for bladder cancer. *European Urology, Supplements*.

[23] Coyne KS, Sexton CC, Irwin DE, Kopp ZS, Kelleher CJ, Milsom I (2008). The impact of overactive bladder, incontinence and other lower urinary tract symptoms on quality of life, work productivity, sexuality and emotional well-being in men and women: results from the EPIC study. *BJU International*.

[24] Lalos O, Berglund AL, Lalos A (2001). Impact of urinary and climacteric symptoms on social and sexual life after surgical treatment of stress urinary incontinence in women: a long-term outcome. *Journal of Advanced Nursing*.

[25] Swithinbank LV, Abrams P (1999). The impact of urinary incontinence on the quality of life of women. *World Journal of Urology*.

[26] Whitmore WF, Marshall VF (1956). Radical surgery for carcinoma of the urinary bladder; one hundred. *Cancer*.

[27] Bochner BH, Montie JE, Lee CT (2003). Follow-up strategies and management of recurrence in urologic oncology bladder cancer: invasive bladder cancer. *Urologic Clinics of North America*.

[28] Mohamed NE, Diefenbach MA (2010). Bladder cancer treatment decision making: results of patients’ interviews. *Annals of Behavioral Medicine*.

[29] Lee CT, Latini DM (2008). Urinary diversion: evidence-based outcomes assessment and integration into patient decision-making. *BJU International*.

[30] Cella DF, Cherin EA (1988). Quality of life during and after cancer treatment. *Comprehensive Therapy*.

[31] Parkinson JP, Konety BR (2004). Health related quality of life assessments for patients with bladder cancer. *Journal of Urology*.

[32] Pyne JM, Sieber WJ, David K, Kaplan RM, Rapaport MH, Williams DK (2003). Use of the quality of well-being self-administered version (QWB-SA) in assessing health-related quality of life in depressed patients. *Journal of Affective Disorders*.

[33] Gilbert SM, Wood DP, Dunn RL (2007). Measuring health-related quality of life outcomes in bladder cancer patients using the Bladder Cancer Index (BCI). *Cancer*.

[34] Ware JE, Sherbourne CD (1992). The MOS 36-item short-form health survey (SF-36). I. Conceptual framework and item selection. *Medical Care*.

[35] Boyd SD, Feinberg SM, Skinner DG, Lieskovsky G, Baron D, Richardson J (1987). Quality of life survey of urinary diversion patients: comparison of ileal conduits versus continent Kock ileal reservoirs. *Journal of Urology*.

[36] Gerharz EW, Weingärtner K, Dopatka T, Köhl UN, Basler HD, Riedmiller HN (1997). Quality of life after cystectomy and urinary diversion: results of a retrospective interdisciplinary study. *Journal of Urology*.

[37] Hart S, Skinner EC, Meyerowitz BE, Boyd S, Lieskovsky G, Skinner DG (1999). Quality of life after radical cystectomy for bladder cancer in patients with an ileal conduit, or cutaneous or urethral Kock pouch. *Journal of Urology*.

[38] Leventhal H, Diefenbach M, Leventhal EA (1992). Illness cognition: using common sense to understand treatment adherence and affect cognition interactions. *Cognitive Therapy and Research*.

[39] Fujisawa M, Isotani S, Gotoh A, Okada H, Arakawa S, Kamidono S (2000). Health-related quality of life with orthotopic neobladder versus ileal conduit according to the SF-36 survey. *Urology*.

[40] Kikuchi E, Horiguchi Y, Nakashima J (2006). Assessment of long-term quality of life using the FACT-BL questionnaire in patients with an ileal conduit, continent reservoir, or orthotopic neobladder. *Japanese Journal of Clinical Oncology*.

[41] Shariat SF, Milowsky M, Droller MJ (2009). Bladder cancer in the elderly. *Urologic Oncology*.

[42] Prout GR, Wesley MN, Yancik R, Ries LAG, Havlik RJ, Edwards BK (2005). Age and comorbidity impact surgical therapy in older bladder carcinoma patients: as population-based study. *Cancer*.

[43] Montie JE (1994). Follow-up after cystectomy for carcinoma of the bladder. *Urologic Clinics of North America*.

[44] Lee CT, Hafez KS, Sheffield JH, Joshi DP, Montie JE (2004). Orthotopic bladder substitution in women: nontraditional applications. *Journal of Urology*.

[45] Gilbert SM, Dunn RL, Hollenbeck BK (2010). Development and validation of the Bladder Cancer Index: a comprehensive, disease specific measure of health related quality of life in patients with localized bladder cancer. *Journal of Urology*.

[46] Cookson MS, Dutta SC, Chang SS, Clark T, Smith JA, Wells N (2003). Health related quality of life in patients treated with radical cystectomy and urinary diversion for urothelial carcinoma of the bladder: development and validation of a new disease specific questionnaire. *Journal of Urology*.

[47] Aaronson NK, Ahmedzai S, Bergman B (1993). The European Organization for Research and Treatment of Cancer QLQ-C30: a quality-of-life instrument for use in international clinical trials in oncology. *Journal of the National Cancer Institute*.

[48] Cella DF (1997). *FACIT Manual: Manual of the Functional Assessment of Chronic Illness Therapy (FACIT) Scales*.

[49] Cella DF, Tulsky DS, Gray G (1993). The functional assessment of cancer therapy scale: development and validation of the general measure. *Journal of Clinical Oncology*.

[50] Somani BK, Gimlin D, Fayers P, N’Dow J (2009). Quality of life and body image for bladder cancer patients undergoing radical cystectomy and urinary diversion–a prospective cohort study with a systematic review of literature. *Urology*.

[51] Mansson A, Johnson G, Mansson W (1988). Quality of life after cystectomy: comparison between patients with conduit and those with continent caecal reservoir urinary diversion. *British Journal of Urology*.

[52] Bjerre BD, Johansen C, Steven K (1995). Health-related quality of life after cystectomy: bladder substitution compared with ileal conduit diversion. A questionnaire survey. *British Journal of Urology*.

[53] Kitamura H, Miyao N, Yanase M (1999). Quality of life in patients having an ileal conduit, continent reservoir or orthotopic neobladder after cystectomy for bladder carcinoma. *International Journal of Urology*.

[54] McGuire MS, Grimaldi G, Grotas J, Russo P (2000). The type of urinary diversion after radical cystectomy significantly impacts on the patient’s quality of life. *Annals of Surgical Oncology*.

[55] Conde Redondo C, Estébanez Zarranz J, Rodríguez Tovez A, Amón Sesmero J, Alonso Fernández D, Martínez Sagarra JM (2001). Quality of life in patients treated with orthotopic bladder substitution versus cutaneous ileostomy. *Actas Urologicas Espanolas*.

[56] Hobisch A, Tosun K, Kinzl J (2001). Life after cystectomy and orthotopic neobladder, versus, ileal conduit urinary diversion. *Seminars in Urologic Oncology*.

[57] Dutta SC, Chang SS, Coffey CS, Smith JA, Jack G, Cookson MS (2002). Health related quality of life assessment after radical cystectomy: comparison of ileal conduit with continent orthotopic neobladder. *Journal of Urology*.

[58] Hara I, Miyake H, Hara S (2002). Health-related quality of life after radical cystectomy for bladder cancer: a comparison of ileal conduit and orthotopic bladder replacement. *BJU International*.

[59] Mansson A, Davidsson T, Hunt S, Mansson W (2002). The quality of life in men after radical cystectomy with a continent cutaneous diversion or orthotopic bladder substitution: is there a difference?. *BJU International*.

[60] Protogerou V, Moschou M, Antoniou N, Varkarakis J, Bamias A, Deliveliotis C (2004). Modified S-pouch neobladder vs ileal conduit and a matched control population: a quality-of-life survey. *BJU International*.

[61] Allareddy V, Kennedy J, West MM, Konety BR (2006). Quality of life in long-term survivors of bladder cancer. *Cancer*.

[62] Saika T, Arata R, Tsushima T (2007). Health-related quality of life after radical cystectomy for bladder cancer in elderly patients with an ileal conduit, ureterocutaneostomy, or orthotopic urinary reservoir: a comparative questionnaire survey. *Acta Medica Okayama*.

[63] Harano M, Eto M, Nakamura M (2007). A pilot study of the assessment of the quality of life, functional results, and complications in patients with an ileal neobladder for invasive bladder cancer. *International Journal of Urology*.

[64] Philip J, Manikandan R, Venugopal S, Desouza J, Javlé PM (2009). Orthotopic neobladder versus ileal conduit urinary diversion after cystectomy—a quality-of-life based comparison. *Annals of the Royal College of Surgeons of England*.

[65] Sogni F, Brausi M, Frea B (2008). Morbidity and quality of life in elderly patients receiving ileal conduit or orthotopic neobladder after radical cystectomy for invasive bladder cancer. *Urology*.

[66] Diefenbach M, Mohamed NE, Turner G, Diefenbach K, Suls JM, Davidson KW, Kaplan RM (2009). Psychcosocial intervention: an overview. *Handbook of Health Psychology*.

[67] McElduff P, Boyes A, Zucca A, Girgis A (2004). *The Supportive Care Needs Survey: A Guide to Administration, Scoring and Analysis*.

[68] Girgis A, Lambert S, Lecathelinais C (2011). The supportive care needs survey for partners and caregivers of cancer survivors: development and psychometric evaluation. *Psycho-Oncology*.

[69] Scherbring M (2002). Effect of caregiver perception of preparedness on burden in an oncology population. *Oncology Nursing Forum*.

[70] The National Cancer Institute (NCI) http://www.cancer.gov/cancertopics/pdq/supportivecare/caregivers/healthprofessional/page1.

